# Nodosin Exerts an Anti-Colorectal Cancer Effect by Inhibiting Proliferation and Triggering Complex Cell Death *in Vitro* and *in Vivo*

**DOI:** 10.3389/fphar.2022.943272

**Published:** 2022-07-22

**Authors:** Huixia Fan, Xiaopeng Hao, Yuan Gao, Jian Yang, Aojun Liu, Yarui Su, Yong Xia

**Affiliations:** ^1^ School of Life Sciences, Henan University, Kaifeng, China; ^2^ Key Laboratory of Precision Oncology of Shandong Higher Education, Institute of Precision Medicine, Jining Medical University, Jining, China; ^3^ School of Pharmacy, Henan University, Kaifeng, China; ^4^ State Key Laboratory Breeding Base of Dao-di Herbs, National Resource Center for Chinese Materia Medica, China Academy of Chinese Medical Sciences, Beijng, China

**Keywords:** nodosin, colorectal cancer, oxidative stress, apoptosis, autophagy

## Abstract

Colorectal cancer (CRC) is one of the most common digestive system cancer in the world. Its incidence and mortality are increasing annually. Presently, CRC lacks long-term effective treatment methods and drugs. Therefore, finding new treatment methods and drugs is of great significance for CRC treatment. Compounds derived from natural plants have been widely used in tumor research and treatment because of their good antitumor activity these years. This study found that nodosin, a diterpenoid extracted from the medicinal plant *Rabdosia serra (Maxim.) Hara,* inhibited the growth of CRC cells SW480, HT-29 and LoVo in a dose- and time-dependent manner, with inhibitory concentrations (IC_50_) of 7.4, 7.7, and 6.6 μM respectively. We selected highly metastatic and poorly differentiated SW480 cells for further studies. We found that nodosin could inhibit cell proliferation by inhibiting DNA synthesis and induce cell death by inducing oxidative stress, apoptosis and autophagy in cells. Through *in vitro* assays combined with transcriptomic analysis, it was found that nodosin could downregulate tribbles pseudokinase 3 and upregulate oxidative stress-induced growth inhibitor 1 to induce oxidative stress in cells; nodosin-induced reactive oxygen species were able to upregulate the expression of heme oxygenase 1 to induce apoptosis and the expression of cathepsin L. and light chain-3 to induce autophagy. *In vivo*, we found that nodosin inhibited tumor growth and induced cells to undergo apoptosis and autophagy without significant toxic effects. In conclusion, our findings suggest that nodosin exerts anti-CRC effects mainly through its ability to induce apoptosis and autophagy *in vitro* and *in vivo*. Therefore, our study contributes to the development of nodosin-based potential CRC therapeutic drugs.

## Introduction

Colorectal cancer (CRC) is a common cancer of the digestive system. Among the common malignant tumors, CRC ranks second in the mortality rate (Keum and Giovannucci, 2019). In recent years, studies have found that an increase in unhealthy eating habits has led to a gradual onset of CRC at younger ages, and CRC incidence has gradually increased among people under the age of 50 (Akimoto et al., 2021). Most patients have progressed to metastatic CRC during diagnosis, and their survival is 1–5 years (Van der Jeught et al., 2018). Current treatments for CRC are limited, and conventional anticancer methods, such as surgery, radiotherapy, and chemotherapy, are highly toxic and nonspecific (Johdi and Sukor, 2020). Cetuximab and panitumumab are often used in clinical practice to treat and extend the survival period of patients (Biller and Schrag, 2021). However, there is no complete cure, and drug resistance emergence is gradually increasing (Talib et al., 2020). Therefore, studying more effective treatment methods and drugs for treating CRC is crucial.

In recent years, natural medicines have been considered essential sources of medicines for treating different diseases. Studies have found that natural compounds can exert anti-proliferative, pro-apoptotic, anti-angiogenic, and anti-mutagenic effects by regulating various biological processes, including the cell epigenome (Samec et al., 2019). In addition, plant-derived compounds can suppress cancer invasion, transfer, and proliferation by regulating various signaling pathways (Sarwar et al., 2018). Therefore, natural compounds have become a relatively widespread antitumor drug for clinical applications. The search for novel antitumor active substances from naturally derived compounds has become a research hotspot for anticancer drugs.

*Rabdosia serra (Maxim.) Hara*, called Xihuangcao in Chinese, is widely used to treat various diseases because of its diverse biological activities, including anti-inflammatory, anti-oxidation, antibacterial, and antitumor activities (Chen et al., 2014). Diterpenoids and phenolics extracted from *Rabdosia serra (Maxim.) Hara* have good medical and health value (Lin et al., 2013). This study found that the diterpenoid nodosin extracted from *Rabdosia serra (Maxim.) Hara* inhibits the activity of CRC cells. Nowadays, most researches on nodosin mainly focus on three aspects: anti-inflammatory, antibacterial, and anticancer activities. In terms of anti-inflammatory activity, nodosin can inhibit mouse ear swelling and serum interleukin-2 (IL-2) levels by regulating the cell cycle of mouse T lymphocytes (Li et al., 2010); In terms of antibacterial activity, the minimal inhibitory concentration 90 (MIC_90_) of nodosin against gram-positive bacteria *Staphylococcus aureus* and *Streptococcus mutans* was 25 μg/ml, and the antibacterial activity was high, while the antibacterial activity against gram-negative bacteria was weak (Isobe and Nagata, 2010); In terms of antitumor activity, nodosin can inhibit the proliferation of CRC HCT116 cells by regulating the Wnt/β-catenin signaling pathway (Bae et al., 2020). However, we found that nodosin had a lower inhibitory concentration (IC_50_) and better anticancer activity against SW480 cells than against HCT116 cells. Moreover, previous studies have been limited to the Wnt/β-catenin signaling pathway, and their effects on animals have not been studied. Therefore, this study aimed to determine the pharmacological mechanism of nodosin in CRC SW480 cells and to verify its effect *in vivo*.

This study conducted an in-depth investigation of the potential anticancer mechanism of nodosin in CRC *in vivo* and *in vitro*. Nodosin was found to inhibit the proliferation, migration, and clonogenicity of SW480 cells by cell counting kit-8 (CCK-8), clone formation, cell migration, 5-ethynyl-2′-deoxyuridine (EdU), and cell immunofluorescence (IF) assays. In addition, combined the transcriptomic analysis results indicated that nodosin could downregulate *tribbles pseudokinase 3* (*TRIB3*) and upregulate *oxidative stress-induced growth inhibitor 1* (*OSGIN1*) to induce oxidative stress in cells. Nodosin-induced reactive oxygen species (ROS) were able to upregulate the expression of *heme oxygenase 1* (*HMOX1*) to induce apoptosis and upregulate the expression of *cathepsin L* (*CTSL*) and *light chain-3* (*LC3*) to induce autophagy. *In vivo*, nodosin inhibited the growth of CRC transplant tumors and induced apoptosis and autophagy through hematoxylin and eosin (H&E) staining and immunohistochemistry assays (IHC). In conclusion, the results of this study will better reveal the anticancer mechanism of nodosin in CRC, provide some ideas and strategies for treating CRC, and help develop nodosin-based anti-CRC drugs.

## Materials and Methods

### Cell Culture

Human CRC SW480, HT-29, Lovo, and NCM460 cells were purchased from American Type Culture Collection (ATCC), and cells were cultured in DMEM medium (06-1055-57-1ACS, BI, Israel), which added 10% Fetal Bovine Serum (FBS, A3160801, Hyclone, United States) and 1% Pecinillin-Streptomycin (P/S, UB89609, GBICO, United States), cultured in 37°C incubation containing 5% CO_2_.

### Chemicals

Nodosin was purchased from Huzhou Zhanshu Biotechnology Co., Ltd. (10391-09-0, molecular formula: C_20_H_26_O_6_, molecular weight: 362.42, purity: 99.98%). DMSO (dimethyl sulfoxide, D8371) was obtained from Solarbio Technology Ltd.

### Cell Viability Assay

Cells with a density of 25% were seeded in 96-well plates, and treated with 0–16 μM nodosin when the total cell density was about 40%. The relative cell activities of SW480, HT-29, LoVo, and NCM460 after nodosin treatment were then detected following the manufacturer’s instruction of CCK-8 kit (CK04, DOJINDO, Japan).

### Cell Morphological Assay

SW480 cells were seeded in the 12-well plates at the confluence of 1 × 10^5^ cells/well. After the cells adhered and returned to normal, the cell morphology at T0 moment was recorded, then treated with 0–12 μM of nodosin for 48 h, and recorded the morphological changes after different times of treatment with an inverted microscope (Nikon, Japan).

### Cell Migration Assay

Before the cells were seeded into 12-well plates, the inserts (80469, IBIDI, Germany) were stuck onto plate bottom following the manufacturer’s instruction. When the cell confluence reached 80%, the insert was removed with forceps to produce a scratch of 500 μm width. The T0 moment was recorded with an inverted microscope (Nikon, Japan). Then added 0–12 μM nodosin and incubated with cells for 24 h, observed and recorded the width of cell scratches with a microscope (Nikon, Japan).

### Cell Membrane Staining Assay

An appropriate amount of cells were seeded in 12-well plates, treated with nodosin for 24 h. Nodosin-treated cells were washed using PBS and fixed with 4% paraformaldehyde for 15 min. After washed 2 to 3 times with PBS, the fixed cells were stained with double dyes (Hoechst 33342 1:1000, C1022, Beyotime; Dio 1:100, C1038, Beyotime) for 20 min, washed twice with PBS, and then photographed with a fluorescence inverted microscope (Nikon, Japan).

### Clone Formation Assay

The cells were seeded in a 12-well plate at a confluence of 500 cells/well. 48 h later, different concentration of nodosin was added. The medium with nodosin was changed every 3 days. After incubation with/without nodosin, the cells were fixed with 4% paraformaldehyde for 20 min at ambient temperature and then stained with crystal violet staining solution (KGA229, KeyGEN BioTECH, China) for 5–10 min. The stained colonies were recorded under an inverted microscope (Nikon, Japan).

### Live/Dead Cell Detection

SW480 cells were seeded in 12-well plates at a number of 1 × 10^5^ cells/well. After the cell density was about 50%, nodosin was added for treatment. The cell viability was detected using a Calcein AM Cell Viability Assay Kit (C2013FT, Beyotime, China), and the stained cells were recorded microscopically (Nikon, Japan).

### Measurement of DNA Synthesis Rate by EdU Method

DNA synthesis in nodosin-treated SW480 cells was detected using EdU-594 Cell Proliferation Assay Reagent (C0078S, Beyotime, China) in the light of the manufacturer’s instruction. The staining results were recorded microscopically (Nikon, Japan), followed by quantitative analysis using ImageJ software.

### Cell Cycle Assay

The nodosin-treated SW480 cells were centrifuged and collected. The cells were resuspended with 70% cold ethanol, fixed on ice for 60 min. After centrifugated at 2,000 rpm, the supernatant was discarded and the pellet cells were resuspended with a mixed solution containing PI (KGA214, KeGEN BioTECH, China), RNase (ST579, Beyotime, China) and Triton X-100 (P0096, Beyotime, China). The stained cells were detected with a flow cytometer (Beckman, cytolfex, United States).

### IF Staining

The cultured cells were fixed with 4% paraformaldehyde at room temperature for 20 min, then permeabilized with 0.3% Triton X-100 for 15 min, blocked with 3% BSA for 30 min. After discarding the block solution, the samples were incubated with Ki67 (1:300, ab15580, abcam, United Kingdom) overnight, and then incubated with CoraLite 488-conjugated Affinipure Goat Anti-Rabbit IgG (H + L) (SA00013-2, proteintech, China) for 1.5 h. The nucleus was stained with DAPI (C1002, Beyotime, China) and the cytoskeleton was stained with Actin-Tracker Red 594 (F-Actin, C2205S, Beyotime, China). The number of the cell proliferation marker Ki67 was observed and recorded with a fluorescence inverted microscope (Nikon, Japan) at a wavelength of 550 nm.

### Detection of ROS by H_2_DCFDA

The changes of ROS in nodosin-induced SW480 cells were detected with the cell-permeable ROS probe H_2_DCFDA (HY-D0940, MCE, United States). SW480 cells in the nodosin-treated and control group were incubated with H_2_DCFDA in a CO_2_ incubator at 37°C for 30 min in the dark. The changes of ROS in the cells were detected by flow cytometry (Beckman, cytolfex, United States). To detect whether ROS was involved nodosin-inhibited cell activity, SW480 cells were pretreated with the antioxidant NAC (ST1546, Beyotime, China) for 1 h, and co-incubated with 12 μM nodosin for 48 h. Finally, the cell activity was detected using the CCK-8 kit.

### Apoptosis Detection

Nodosin-treated SW480 cells were digested with EDTA-free trypsin (T1350, Solarbio, China). The cells were incubated with Annexin V-FITC (C1062M, Beyotime, China) and PI (KGA214, KeyGEN BioTECH, China) for 20 min in the dark. The rate of apoptosis was determined and recorded by flow cytometry (Beckman, cytolfex, United States).

### Plasmid Transfection

Lipofectamine 3000 Transfection Kit (L3000015, Invitrogen, United States) was used to transfer lamp1 OFP, ER, and mito plasmids (gifts from Camilla Raiborg, University of Oslo, Norway) into SW480 cells. After the target plasmids expressed, different concentrations of nodosin were added. Then the confocal laser microscopy (Leica TCS SP8, Leica, Germany) was used to examine the changes of intracellular lysosomes, endoplasmic reticulum, and mitochondria.

### Autophagy Detection

SW480 cells were cultured in a 96-well plate at 37°C with 5% CO_2_ for 12 h. Next, they were treated with 12 μM of nodosin for 48 h. SW480 cells were then incubated with 6.25–250 nM of the autophagy blocker chloroquine (CQ, HY-17589A, MCE, United States) for 2 h, and cell viability was detected using the CCK-8 kit.

### Transcriptome Assay

In transcriptome sequencing, three nodosin-treated samples and three control samples were lysed with Trizol reagent (15596026, ambition, Japan) respectively (5 × 10^6^ cells in each sample). Library construction, mRNA sequencing, and bioinformatics analysis were performed at BIOMARKER (Beijing, China).

### Reverse Transcriptional PCR and Real-Time PCR

Total RNA in cells was extracted with Trizol reagent (15596026, ambition, Japan), then RNA was reversed into cDNA with FastKing RT Kit (With gDNase) (KR116, TIANGEN, China), validated with agarose gel, and then qPCR was conducted using SuperReal PreMix Color (SYBR Green, FP215, TIANGEN, China), relative mRNA levels were normalized to β-actin levels, and relative gene expression changes were calculated. The primers of PCR and q-PCR are shown in Tables 1, 2.

**TABLE 1:** The primer sequences for PCR.

Primer	PCR primer
HMOX1	Forword: CAG​GCA​GAG​AAT​GCT​GAG​T
	Reverse: TTGAACTTGGTGGCACTG
CTSL	Forword: TCC​TAC​ACT​CAT​CCT​TGC​TG
	Reverse: AAC​CAC​ACT​GAC​CCT​GAT​TC
β-actin	Forword: CAC​TCT​TCC​AGC​CTT​CCT​T
	Reverse: ACAGGTCTTTGCGGATGT

**TABLE 2:** The primer sequences for realtime-PCR.

Primer	Quantitative RT-PCR primer
HMOX1	Forword: ATTGCCAGTGCCACCAAGT
	Reverse: TGAGCAGGAACGCAGTCTT
CTSL	Forword: TCCTACACTCATCCTTGCTG
	Reverse: TCCACTGTGCCTCTAAACTG
β-actin	Forword: CACTCTTCCAGCCTTCCTT
	Reverse: ACAGGTCTTTGCGGATGT

### Western Blotting

The nodosin-treated SW480 cells were lysed and proteins were extracted from them using lysis buffer (P0013, Beyotime, China). The protein extracts were processed by SDS polyacrylamide gel electrophoresis and blotted on PVDF membranes. Western blotting (WB) was performed using the following primary antibodies: HMOX1 (Rabbit, 1:3,000, 10701-1-AP, proteintech, China), LC3 (Rabbit, 1:2,000, 14600-1-AP, proteintech, China), β-Actin (Rabbit, 1:2,000, GB11001, Servicebio, China). After the primary antibody was incubated and washed, the immunohy bridization signal was captured using HRP-conjugated secondary antibody. The secondary antibodies were listed as follows: HRP Goat Anti-Rabbit IgG (H + L) (1:4,000, AS014, ABclonal, China). The development was then performed using ECL solution (P0018S, Beyotime, China).

### Xenograft in Nude Mice

BALB/c-nude nude mice were provided by Jinan Pengyue Laboratory Animal Breeding Co.,Ltd., eight females, 6 weeks old, about 20 g, SPF grade, the laboratory animal certificate number was No. 370726211100958867. This assay was performed with the approval of the Ethics Committee of Jining Medical University. BALB/c-nude female mice were subcutaneously injected with CRC SW480 cells. The mice were randomly divided into two groups (4 nude mice/group) when the tumor grew to 100 mm^3^. In the nodosin-treated group, the mice were treated by nodosin with a dose of 3 mg/kg, once every 3 days. The control group was treated with DMSO at the same injection volume and frequency. After 10 injections, the CRC xenograft tumors of mice were removed, weighed and photographed. Paraffin blocks were made after the tumors were fixed with 10% neutral formalin and dehydrated for subsequent studies.

### H&E Staining

The CRC xenograft tumors paraffin blocks were cut into slices (the thickness was 4 μm). The tumor sections were stained in the light of the instructions of the HE staining kit (G1120, Solarbio, China). The changes of the pathological sections were observed under a Pannoramic Desk (3DHISTECH, Hungary).

### IHC Staining

The biomarkers in nude mice transplanted tumors were detected by IHC methods. Cut the wax block into 4-μm wax slices, and use an oven at 60°C for 1–2 h. The sections were removed for dewaxing, hydration, and then antigen repair with improved sodium citrate antigen repair solution (P0083, Beyotime, China). Incubated for 30 min with inactivated endogenous peroxidase 3% H_2_O_2_ (SV0004, BOSTER, China) to avoid light. Afterwards, they were blocked with ready-to-use normal goat serum (AR0009, BOSTER, China) for 30 min. After shaking off the blocking solution, Ki67 (1:300, ab15580, abcam, United Kingdom), HMOX1 (Rabbit, 1:200, 10701-1-AP, proteintech, China), LC3 (Rabbit, 1:500, 14600-1-AP, proteintech, China), CTSL (Rabbit, 1:500, proteintech, China) were added and placed in a refrigerator at 4°C overnight for incubation. Polymeric HRP-labeled anti-rabbit/mouse IgG (SV0004, BOSTER, China) was added after washing with PBS and incubated for 30 min at room temperature in the dark. The color was developed with DAB chromogen solution (C02-12, ORIGENE, America). After color development, counterstain with hematoxylin (AR0005, BOSTER, China). Afterwards, it was blue with alkaline PBS. Finally, dehydration was performed and the slides were mounted. Scanning observations were performed using a Pannoramic Desk (3DHISTECH, Hungary).

### Statistics

The difference between the two groups was assessed by two-tailed *t*-test. The three groups and above were evaluated with one-way ANOVA analysis followed by LSD Post Hoc multiple comparison to assess its significance, and *p*-value < 0.05 was considered significant.

## Results

### Nodosin Repressed CRC Cells in a Dose- and Time-Dependent Way *in vitro*

**FIGURE 1:**
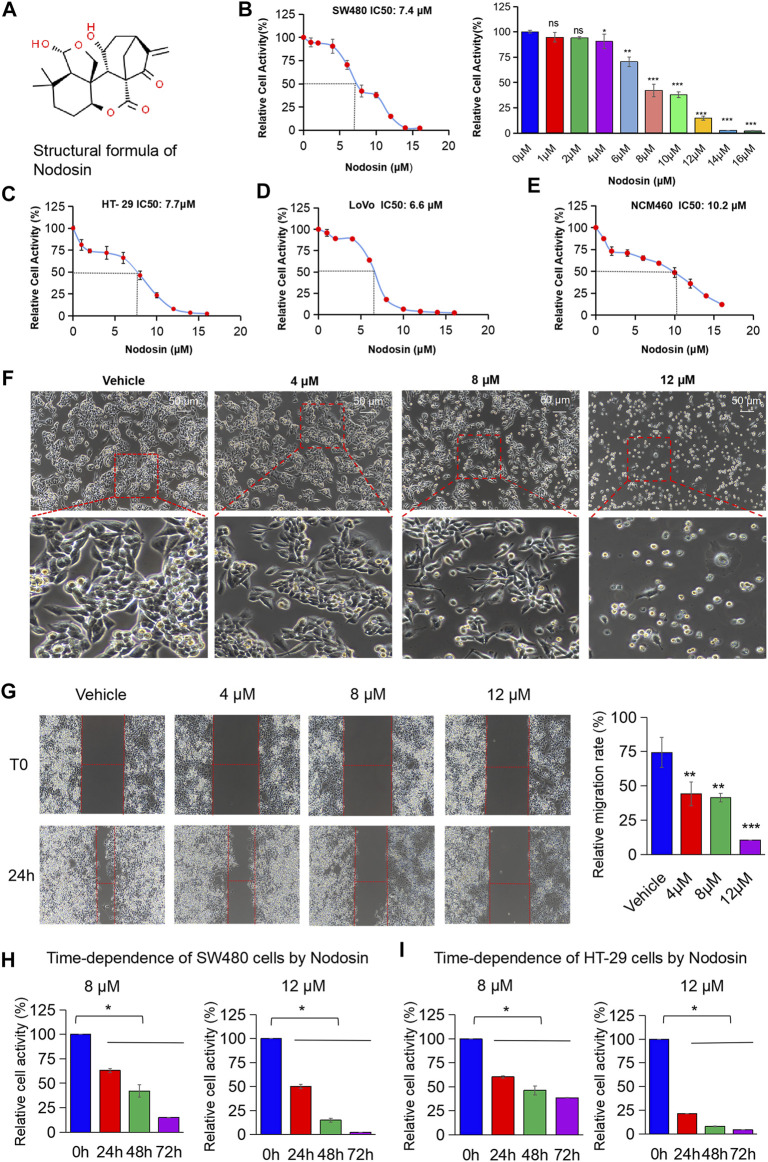
Nodosin suppressed CRC cells in dose- and time-dependent way *in vitro*. **(A)** The chemical structural formula of nodosin (http://www.chemspider.com). **(B)** CRC SW480 cells were treated with 0–16 μM nodosin for 48 h. The relative viability of cells was detected by CCK-8 kit. Each group was repeated three times. The significant difference between the nodosin treated and control group (without nodosin) was evaluated by the two-tailed *t*-test, *p < 0.05, **0.01 < *p* < 0.05, ***p < 0.01. **(C)** HT-29 cells were treated with 0–16 μM nodosin for 48 h. The cell viability was tested by CCK-8 kit. **(D)** LoVo cells were treated with 0–16 μM nodosin for 48 h. The cell viability was tested by CCK-8 kit. **(E)** NCM460 cells were treated with 0–16 μM nodosin for 48 h. The cell activity was tested by CCK-8 kit. **(F)** SW480 cells were treated with nodosin for 48 h, and the morphological changes of SW480 cells were recorded by fluorescence inverted microscope. **(G)** The repressive effect of nodosin on the migration of SW480 cells was tested by cell scratch method. SW480 cells were treated with different concentrations of nodosin for 24 h to measure the scratch width and calculate the cell scratch closure rate. The relative migration rate significant difference between the nodosin treated group and the control group (without nodosin) was evaluated by the two-tailed *t*-test, *p < 0.05, **p < 0.01, ***p < 0.001. **(H)** Nodosin reduced the relative activity of SW480 cells in a dose- and time-dependent manner. The significant difference was evaluated with one-way ANOVA analysis followed by LSD Post Hocnultiple comparison, and *p*-value < 0.05 was considered significant. **(I)** Nodosin reduced the relative activity of HT-29 cells in a dose-dependent and time-dependent way. The significance difference was evaluated with one-way ANOVA analysis followed by LSD Post Hocnultiple comparison, and *p*-value < 0.05 was considered significant.

The chemical structure of nodosin is shown in Figure 1A. According to the CCK-8 assay results (Figures 1B–D), nodosin significantly repressed CRC cells proliferation in a dose-dependent manner. The IC_50_ of nodosin in SW480, HT-29, and LoVo cells were 7.4, 7.7, and 6.6 μM, respectively. However, the toxicity to human normal colonic mucosal epithelial cells NCM460 was lower, with an IC_50_ of 10.2 μM (Figure 1E). In addition, the morphological characteristics of CRC SW480 cells were significantly changed after nodosin treatment (Figure 1F). At low concentrations, the cells underwent significant shrinkage and elongation; at high concentrations, the cells were unable to adhere and became rounded. Figure 1G shows that nodosin dose-dependently restrained the migration ability of SW480 cells. Cell mobility decreased gradually with increased nodosin concentration, and almost no cell migration occurred at 12 μM. Therefore, the relative activity of cells gradually decreased with increased nodosin concentration and prolonged treatment (Figures 1H,I), indicating that nodosin could inhibit the proliferation of CRC cells in a dose- and time-dependent manner.

### Nodosin Suppressed CRC Cells Proliferation and Triggered Cells Death

**FIGURE 2:**
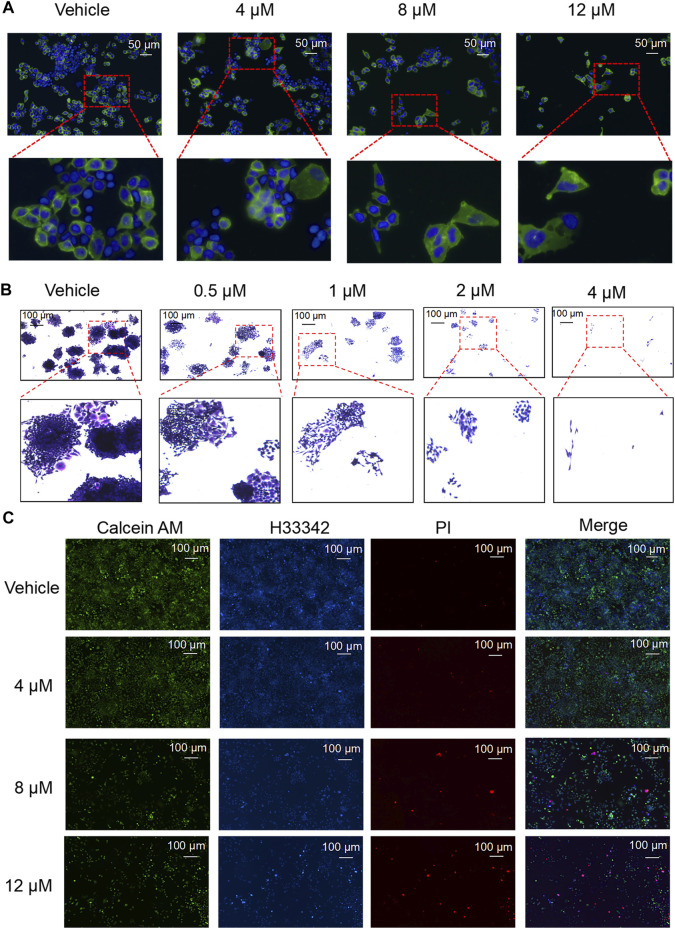
Nodosin supressed cell proliferation and triggered cell death. **(A)** Nodosin-treated SW480 cells were stained with Dio and Hoechst 33342 (Dio stained membranes in green and Hoechst 33342 stained nucleus in blue). **(B)** Different concentrations of nodosin treated SW480 cells for 12 days. Crystal violet stained cell communities were observed with an inverted microscope. **(C)** Nodosin-treated SW480 cells were stained with Hoechest 33342, Calcein AM and PI. The stained results were recorded by fluorescence inverted microscope (Hoechest 33342 stained cells in blue, Calcein AM stained cells in green and PI stained cells in red).

Proliferation and death are important processes in the development of tumor cells. As nodosin concentration increased, the number of cells gradually decreased, and the cell membrane became larger and damaged (Figure 2A). Additionally, nodosin inhibited the clonogenic ability of SW480 cells. When nodosin concentration was greater than 4 μM, the cells could not form clonal colonies (Figure 2B). Interestingly, nodosin only inhibited 10% of cell activity at a concentration of 4 μM, whereas nodosin at the same concentration inhibited majority of the clonogenic ability of SW480 cells. Moreover, the number of live cells decreased, and the number of dead cells gradually became elevated with increased nodosin concentrations in the live/dead cell staining assay (Figure 2C).

### Nodosin Inhibited DNA Synthesis Rate in CRC Cells

**FIGURE 3:**
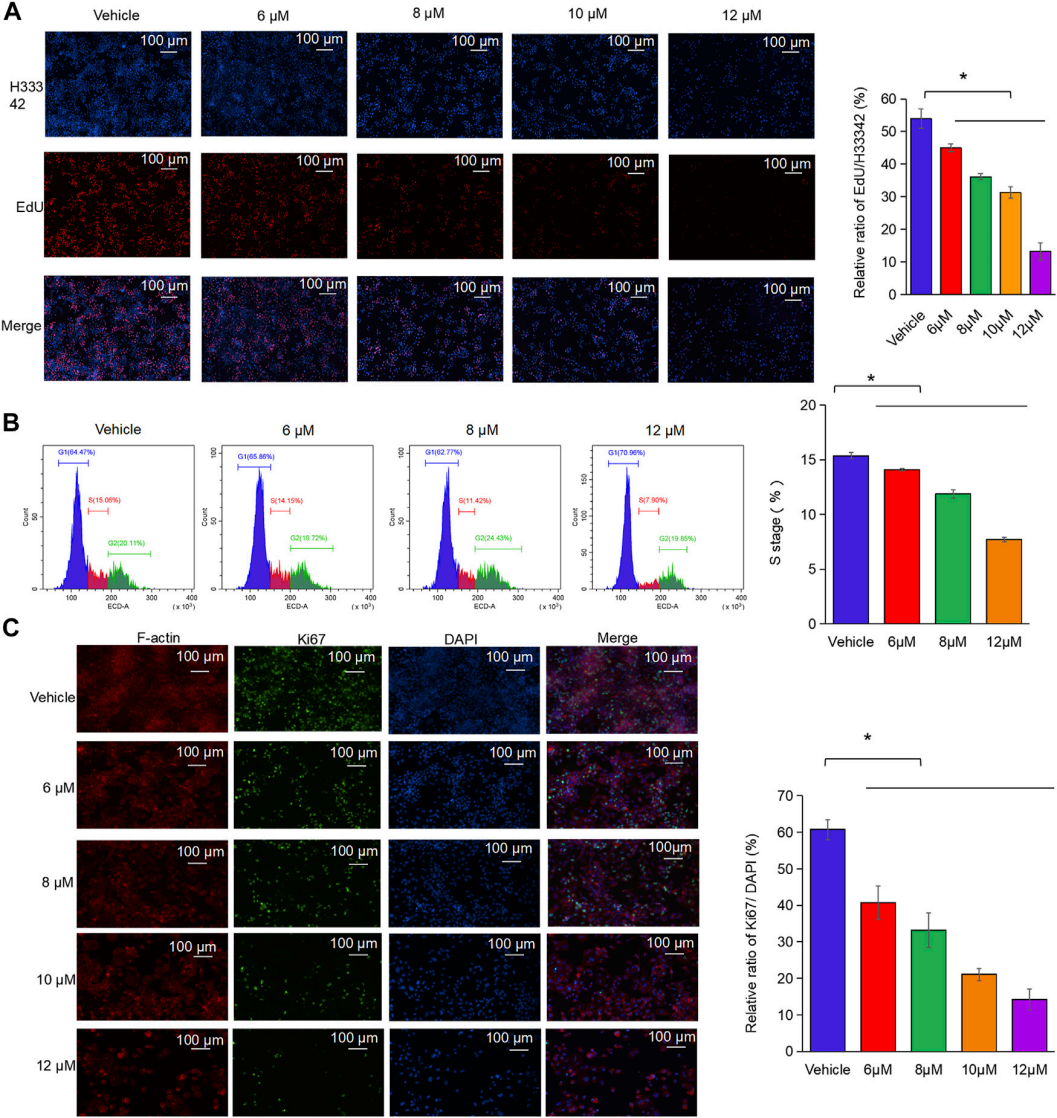
Nodosin repressed the proliferation of CRC cells by inhibiting DNA synthesis. **(A)** Nodosin-treated SW480 cells were stained with EdU and Hoechst 33342, and then cell proliferation was observed with fluorescence inverted microscope (EdU stained cells in red, Hoechst 33342 stained cells in blue). **(B)** Nodosin-treated SW480 cells were stained with PI. The cell cycle of staining cells was detected by flow cytometry. The blue area was G1 phase cells, the red area was S phase cells, and the green area was G2 phase cells. The significant difference was evaluated with one-way ANOVA analysis followed by LSD Post Hocmultiple comparison, and *p*-value < 0.05 was considered significant. **(C)** SW480 cells were treated with different concentrations of nodosin for 24 h. The Ki67 was detected by IF. F-actin was red fluoreacence, Ki67 was green fluorescence, and DAPI was blue fluorescence. The significance difference was evaluated with one-way ANOVA analysis followed by LSD Post Hocmultiple comparison, and *p*-value < 0.05 was considered significant.

DNA replication speed is an important indicator for evaluating the cell proliferation rate, activity, and physiological status. This study found that nodosin inhibited DNA replication ability of SW480 cells by EdU assay (Figure 3A). After nodosin treatment, the total number of cells decreased to a certain extent. The relative ratio of EdU/H33342 gradually decreased as the nodosin concentration increased, which indicated that nodosin effectively inhibited the DNA replication ability of SW480 cells. Moreover, the cell cycle of SW480 cells was detected after nodosin treatment, and nodosin affected the synthesis (S) phase of the cell cycle; the S phase gradually decreased with increased nodosin concentration (Figure 3B). Ki67 is an antigen related to cell proliferation and exerts an important role in mitosis. In Ki67 immunofluorescence staining (Figure 3C), the Ki67-labeled antigen gradually decreased with an increase in nodosin treatment concentrations, which is consistent with the experimental results shown in Figures 3A,B. These results demonstrate that nodosin can inhibit DNA synthesis from multiple perspectives.

### Nodosin Induced Oxidative Stress, Apoptosis, and Autophagy in SW480 Cells

**FIGURE 4:**
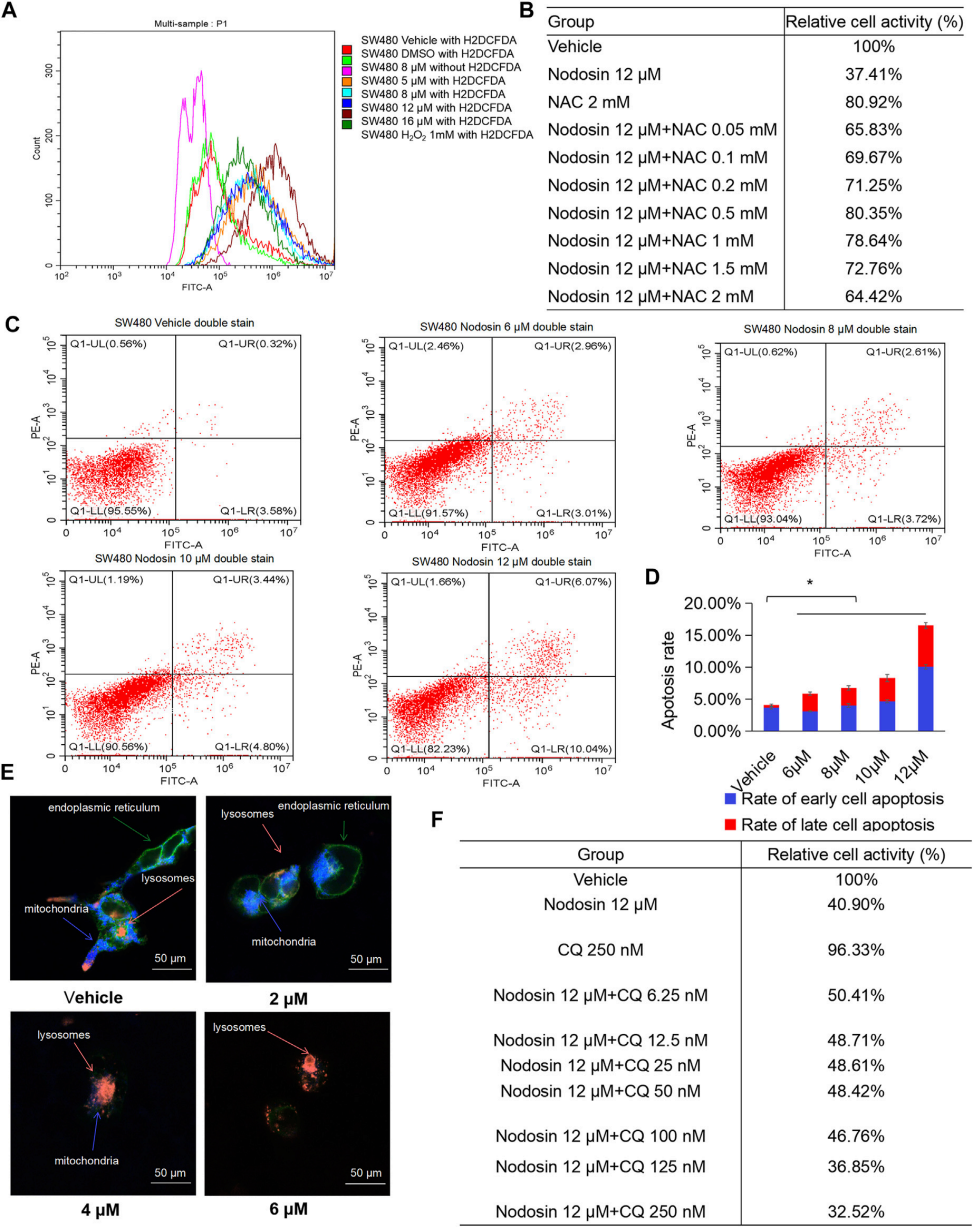
Nodosin triggered cell death in CRC in a manner of oxidative stress, apoptosis and autophagy. **(A)** Intracellular ROS changes in SW480 caused by nodosin were determined by H_2_DCFDA, red indicated the blank control (no ROS probe), light green indicated the solvent control (DMSO treatment, including the ROS probe), purple indicated the negative control (8 μM nodosin treatment, but with no ROS probe available), orange indicated the 5 μM nodosin treated and containing the ROS probe, light blue indicated the 8 μM nodosin treated and containing the ROS probe, dark blue indicated the 12 μM nodosin treated and containing the ROS probe, brown indicated the 16 μM nodosin treated and containing the ROS probe, dark green indicated a positive control (treated with 1 mM H_2_O_2_). **(B)** SW480 cells were pretreated with ROS inhibitor NAC for 1 h and treated with nodosin for 48 h. The relative viability of cells was detected by CCK-8 kit. **(C)** Flow cytometry was employed to determine the apoptosis rate. X axis represented FITC intensity of Annexin-V on the extracellular membrane, Y axis represented PI staining intensity of cells, early apoptotic cell populations were in Q1-LR, late apoptotic cell populations were in Q1-UR. **(D)** The rate of SW480 cells apoptosis. The significant difference was evaluated with one-way ANOVA analysis followed by LSD Post Hocmultiple comparison, and *p*-value < 0.05 was considered significant. **(E)** SW480 cells (transfected with lamp1 OFP, ER, and mito) were treated with different concentrations of nodosin. The intracellular changes in lysosomes, endoplasmic reticulum, and mitochondria were recorded by laser confocal microscopy. Orange represented lysosomes, blue represented mitochondria, and green representsed endoplasmic reticulum. **(F)** After SW480 cells were treated with nodosin for an appropriate period, different concentrations of chloroquine were added to rescue the nodosin-inactivated cells with the CCK-8 kit.

To further study the antitumor mechanism of nodosin, H_2_DCFDA fluorescent probe was used to test intracellular ROS levels after nodosin treatment. Figure 4A shows that after the cells were stimulated by nodosin, oxidative stress occurred, and the ROS level in the cells increased continuously with an increase in nodosin treatment concentration. In this regard, after pretreatment with the antioxidant N-acetylcysteine (NAC), the relative cell activity reached saturation when the NAC concentration was 0.5 mM, and the relative cell activity could be increased to 80%, indicating that nodosin induced oxidative stress-mediated cell death. The evidence that pretreatment with NAC reversed oxidative stress-induced changes in SW480 cell death validated the dependence on oxidative stress (Figure 4B). Oxidative stress may lead to cell apoptosis and autophagy; therefore, an apoptosis assay using flow cytometry was performed. These results revealed that nodosin induced cell apoptosis. The apoptosis rate gradually elevated with increased nodosin concentration. When the concentration of nodosin was 12 μM, the apoptosis rate of the cells was 16.11% (Figures 4C,D). Therefore, we speculated that there are other modes of cell death in addition to apoptosis. Elevated levels of ROS usually lead to stress in mitochondria, endoplasmic reticulum, and lysosomes; therefore, the changes in these cell organelles after nodosin treatment were observed by plasmid transfection assay. This study’s findings showed that mitochondria and endoplasmic reticulum gradually disappeared with increased nodosin concentration, while lysosomes gradually increased. The endoplasmic reticulum and mitochondria almost completely disappeared at a nodosin concentration of 6 μM (Figure 4E). Based on this phenomenon, we hypothesized that these cells may have undergone autophagy. To verify whether nodosin can induce autophagy in cells, the autophagy blocker chloroquine was used to rescue nodosin-treated SW480 cells. Chloroquine rescued nodosin-induced SW480 cells cell death to a certain extent. At a chloroquine concentration of 6.25 nM, the relative cell activity increased by approximately 10%. These results illustrate that nodosin induced autophagy in SW480 cells (Figure 4F).

### Transcriptomic Analysis of Nodosin Inhibition of CRC Tumors

**FIGURE 5:**
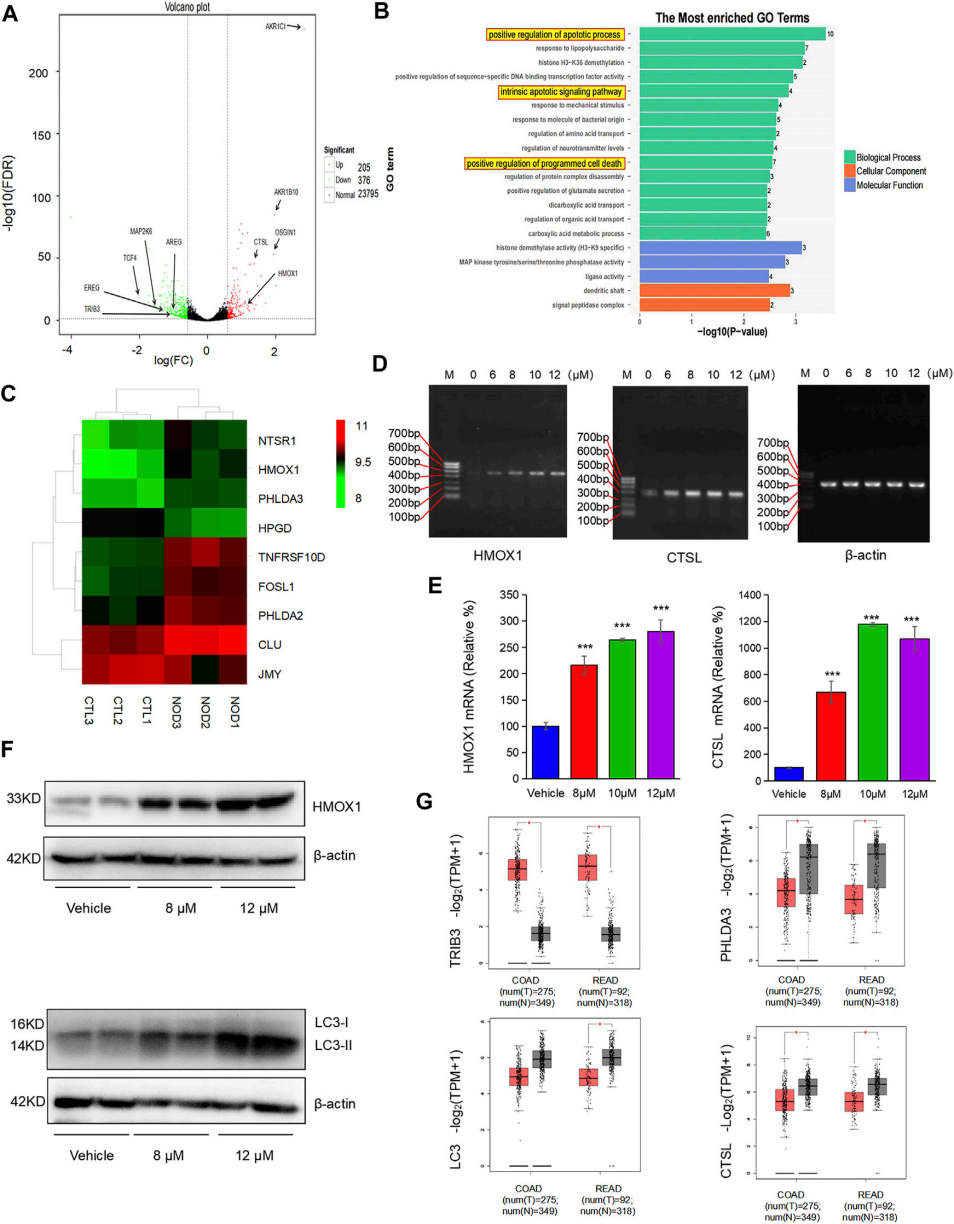
Transcripptomic results demonstrated that nodosin induced oxidative stress, apoptosis, and autophagy. **(A)** Voclano plot of the differential genes between the nodosin treated and control groups. **(B)** GO analysis enriched for biological processes with more obvious differential gene changes, with more differential genes in biological processes related to apoptosis. **(C)** Heatmap of the differential genes associated with apoptosis between the nodosin treated and control groups. **(D)** The cDNA levels of *HMOX1*, *CTSL*, and *β-actin* were measured by PCR. **(E)** The mRNA levels of *HMOX1*, *CTSL*, and *β-actin* were detected by q-PCR. Each test was repeated three times, and the difference was tested by a two-tailed *t*-test. *p < 0.05, **p < 0.01, ***p < 0.001. **(F)** The protein levels of HMOX1, β-actin, and LC3 were tested by western blotting. **(G)** Expression of the oxidative stress, apoptotic and autophagy-related genes *TRIB3*, *PHLDA3*, *LC3*, and *CTSL* in clinical CRC and normal tissues (http://gepia.cancer-pku.cn/index.html, COAD: Colon adenocarcinoma; READ: Rectal adenocarcinoma, *p < 0.05).

To investigate the anticancer mechanism of nodosin, transcriptomic sequencing was performed. Figure 5A shows a volcano plot of differentially expressed genes (DEGs) between the nodosin treatment and control groups. With nodosin treatment, 205 genes were upregulated, and 376 genes were downregulated. Among them, *aldo-keto reductase family 1 member C1* (*AKR1C1*), *aldo-keto reductase family 1 member B10* (*AKRIB10*), *OSGIN1*, *HMOX1*, and *CTSL* were significantly upregulated, whereas *transcription factor 4* (*TCF*4), *TRIB3*, *syntrophin beta 1* (*SNTB1*), *kinase family member 21B* (*KIF21B*), *mitogen-activated protein kinase 6* (*MAP2K6*), *cadherin 11* (*CDH11*), *amphiregulin* (*AREG*), and *epiregulin* (*EREG*) were significantly downregulated. Most of these genes were involved in signaling pathways related to cell proliferation inhibition and cell death induction. Figure 5B shows that gene ontology (GO) analysis was enriched in biological processes with more significant DEGs changes. We drew a heatmap of the DEGs involved in apoptosis and death. *HMOX1* was also upregulated (Figure 5C). These changes induced oxidative stress in the cells, which in turn induced apoptosis. To verify the high-throughput sequencing results, specific primers were designed for verification at the messenger RNA (mRNA) level. The results of PCR and qPCR showed that the *HMOX1* and *CTSL* levels, which are related to apoptosis and autophagy, were significantly increased (Figures 5D,E). Furthermore, WB was performed to verify whether nodosin affected the above pathways at the protein level. The results showed that the apoptosis-and autophagy-positive relative molecules HMOX1 and LC3 were significantly elevated (Figure 5F). Since the transcriptomic results indicated that nodosin affected the expression of genes related to oxidative stress, apoptosis, and autophagy in CRC cells, we validated it using the commonly used gene expression profiling interactive analysis (GEPIA) database in clinical practice. The results revealed that the gene TRIB3 negatively associated with oxidative stress was significantly downregulated, the genes *PHLDA3*, *LC3*, and *CTSL* related to apoptosis and autophagy were significantly upregulated in normal colorectal tissues compared to colorectal tumors (Figure 5G), while nodosin also reversed the expression of these genes, indicating that nodosin is beneficial for the treatment of CRC.

### Nodosin Suppressed the Growth of CRC Xenografted Tumors *in Vivo*

**FIGURE 6:**
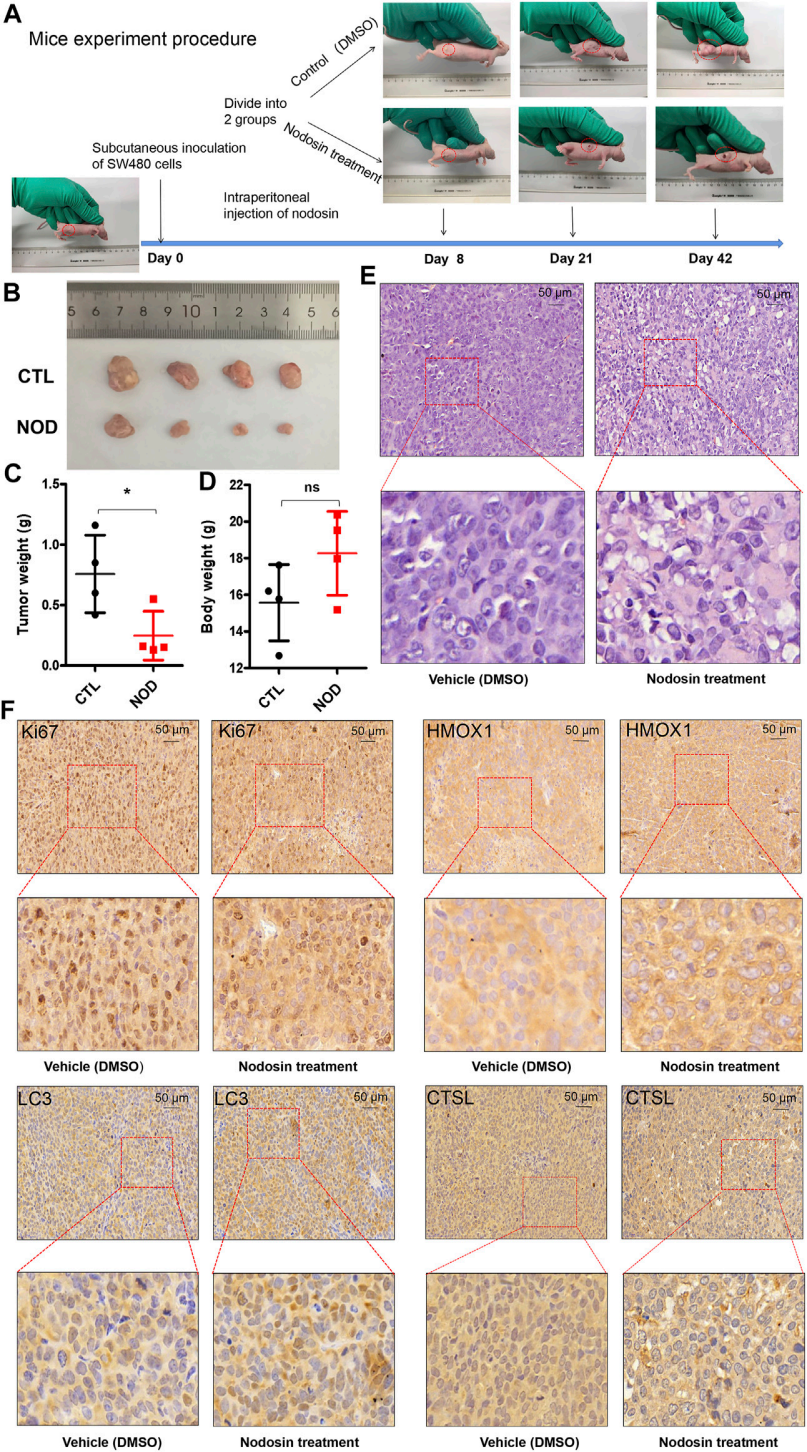
Nodosin supressed the growth of CRC xenograft tumors in nude mice. **(A)** The whole procedure of the nude mice experiment. **(B)** The xenograft tumors isolated from nude mice. **(C)** Tumor weights were compared in nude mice from nodosin treated and control groups. **(D)** Body weight was compared from nodosin treated versus control groups. **(E)** The nodosin-treated and control xenograft tumors were stained with H&E. **(F)** IHC was used to detect the expression of Ki67, HMOX1, LC3, and CTSL in nodosin-treated and control group, and photographed under microscope.

To investigate the antitumor effect of nodosin on CRC *in vivo*, nude mouse subcutaneous tumorigenesis models were used for administration. Figure 6A shows the growth process of xenografted tumors in nude mice. Tumors in the nodosin-treated group grew slower than those in the control group. After the mice were terminated, tumors were isolated and photographed (Figure 6B). When the tumors were weighed, there was a significant difference in tumors weight between the treatment and control group (Figure 6C). There was no significant change in the bodyweight of nude mice in the two groups (Figure 6D), indicating that nodosin had no obvious side effects. H&E and IHC stainings of the pathological tumor sections were performed. H&E staining showed that compared with the control group, the cell structure of the tumor was destroyed, and the number of nuclei was also reduced (Figure 6E). The results of IHC staining revealed that the level of proliferation marker Ki67 was significantly lower in the nodosin-treated group than in the control group, indicating that treatment with nodosin could supress the proliferation of colorectal xenografted tumors. In addition, the expression of the apoptosis marker HMOX1 and the autophagy markers LC3 and CTSL were significantly increased in the nodosin-treated group, which indicated that nodosin could induce apoptosis and autophagy in colorectal tumor cells (Figure 6F).

## Discussion

It is difficult to completely cure CRC due to its high recurrence, easy metastasis, short survival time, limited treatment, and easy drug resistance emergence, which has become a challenging problem in the medical community (Vaghari-Tabari et al., 2020). The increasing incidence of CRC and the trend toward a younger onset have forced researchers to develop new drugs to replace existing drugs (Stoffel and Murphy, 2020). In recent years, natural plant-derived compounds have been found to target various signaling pathways with good tolerance and few toxic side effects (Rejhova et al., 2018). The combination of natural compounds and traditional chemotherapeutic drugs can be considered to provide new directions for the research and treatment of cancer.

Using cell and animal models, this study discovered that nodosin, a diterpenoid extracted from *Rabdosia serra (Maxim.) Hara* has antitumor activity against CRC. Furthermore, combined transcriptomic analysis revealed that nodosin could promote SW480 cell death through multiple pathways, including oxidative stress, apoptosis, and autophagy.

Oxidative stress and the resulting oxidative damage are important factors in tumorigenesis and progression (Klaunig 2018). Basak et al. (2020) found that oxidative stress is closely associated with the occurrence and development of CRC and that oxidative stress caused by excessive production of ROS can selectively eradicate tumor cells. This study found that nodosin increased ROS levels in CRC SW480 cells (Figure 4A). After the rescue of the antioxidant NAC, the activity of the cells significantly increased, indicating that the cells underwent oxidative stress (Figure 4B). *TRIB3*, a modulator of cellular responses to various stresses, is upregulated in various cancers (Tang et al., 2020). *TRIB3* expression in colorectal tumors was also higher compared to normal colorectal tissues in the GEPIA database (Figure 5G). This study found that nodosin could downregulate *TRIB3* expression, implying that nodosin may induce oxidative stress in cells. In addition, excessive ROS production can lead to oxidative stress and programmed cell death, such as apoptosis and autophagy (Gao et al., 2020). Tsai et al. (2017) found that the upregulation of *OSGIN1* could elevate ROS levels, activate PI3K/Akt/Nrf2 signaling, and trigger apoptosis in cells. Wang et al. (2017) found that OSGIN1 could induce autophagy-positive related molecules, such as LC3, at the protein and mRNA levels. Interestingly, our study found nodosin upregulated the expression of *OSGIN1* and *LC3*. Therefore, we speculated that nodosin was able to trigger oxidative stress, apoptosis, and autophagy in SW480 cells by increasing cellular ROS levels.

The transcriptomic analysis revealed that nodosin was able to activate intrinsic and extrinsic apoptotic signaling pathways in SW480 cells (Figure 5B). *HMOX1* was upregulated in the apoptotic signaling pathway. *HMOX1* triggered heme breakdown, releasing reactive oxide species and enhancing intercellular oxidative stress-induced apoptosis (Chiang et al., 2019). PCR, RT-PCR, WB, and IHC results showed that nodosin upregulated the HMOX1 expression, indicating that nodosin induced cell death in an *HMOX1* modulation-related manner. These results suggested that the cell death-promoting mechanism of nodosin was to induce apoptosis by upregulating *HMOX1*. Moreover, Gandini et al. (2019) found that upregulation of *HMOX1* could inhibit the growth of breast cancer tumors and prolong patient survival. Therefore, we considered the development of nodosin-based apoptosis inducers to induce CRC cells death.

ROS induces apoptosis and autophagy in cells. Autophagy is another programmed cell death pathway in addition to apoptosis. It has been reported that autophagy can kill anti-apoptotic cells (Li et al., 2017). In addition, autophagy responds to stressful conditions, including oxidative stress (Zhou et al., 2021). In different types of autophagy, lysosomes can degrade and remove oxidized/damaged proteins and large organelles that produce ROS (such as mitochondria and peroxisomes) (Mahapatra et al., 2021). Through co-transfection with three markers labeled endoplasmic reticulum, mitochondria, and lysosomes, we found that nodosin could cause the endoplasmic reticulum and mitochondria to vanish but increase lysosomes in a dose-dependent manner (Figure 4E). More importantly, nodosin-induced cell inactivation was significantly rescued by the autophagy blocker chloroquine, suggesting that autophagy may be an essential pathway in nodosin-triggered cell death (Figure 4F). Presently, markers related to autophagy mainly include CTSL and LC3. *CTSL*, which encodes lysosomal cysteine protease, promotes autophagic flux (Thirusangu et al., 2021). Han et al. (2016) found that upregulation of CTSL enhances the efficacy of anticancer drugs against lung cancer and other types of malignant tumor cells. Furthermore, the expression of another key autophagy-related protein, LC3, was significantly increased following nodosin treatment. LC3 plays a crucial role in autophagosome formation and autophagy development (Song et al., 2019). This study demonstrated that nodosin could upregulate the expression of CTSL (Figures 5D–F), suggesting that the cell death-promoting mechanism of nodosin-modulated CTSL, which in turn, caused autophagy in CRC cells. See et al. (2018) found that upregulation of the autophagy marker LC3 promotes autophagy and induces CRC cell death. Therefore, targeting autophagy-related proteins may be an effective treatment strategy for CRC.

**FIGURE 7:**
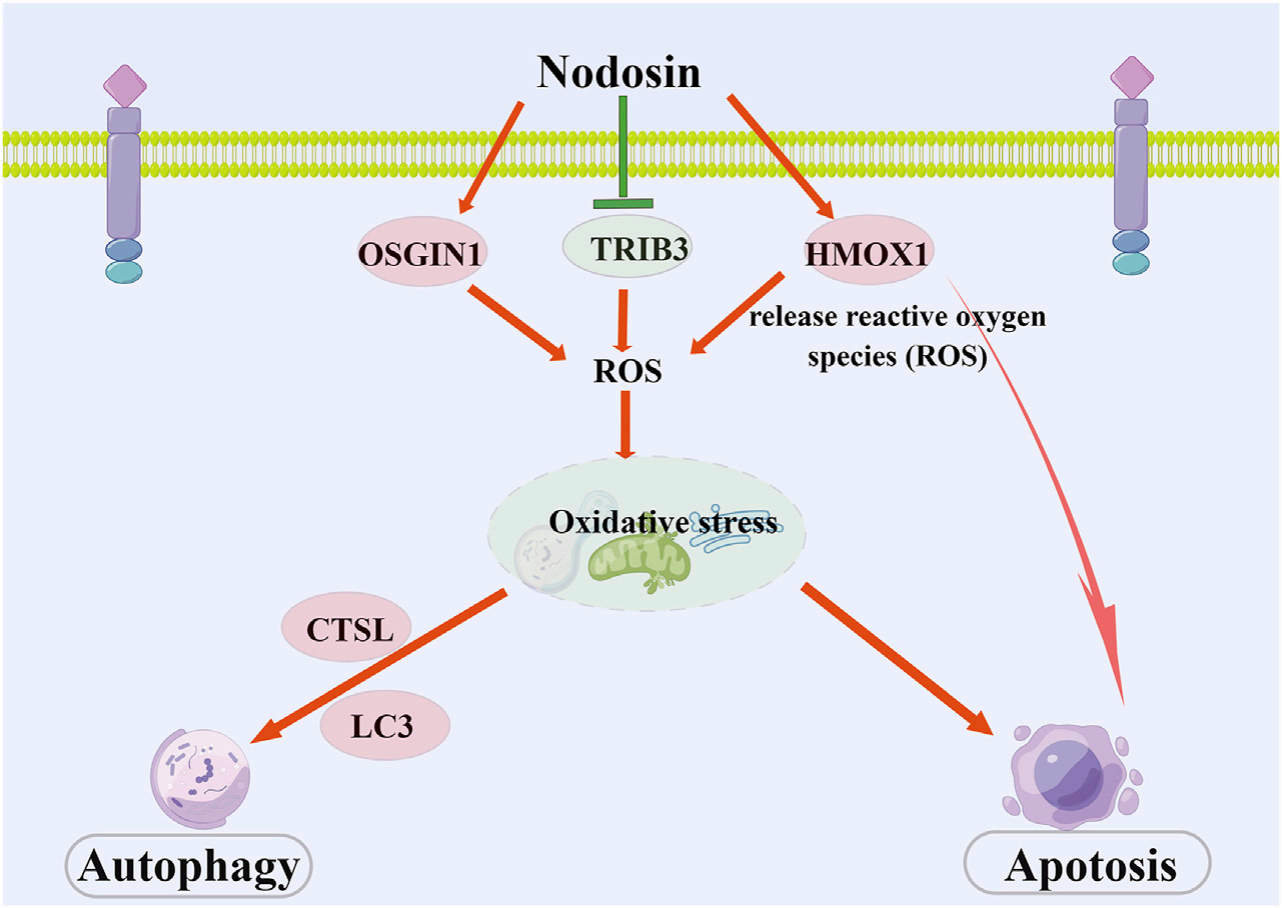
The schematic diagram of pharmacological mechanism of nodosin against CRC. Nodosin inhibited CRC mainly by triggering oxidative stress, apoptosis, and autophagy. The observed pharmacological mechanism of action of nodosin on CRC is as follows: 1) Nodosin could downregulate TRIB3 and upregulate OSGIN1 to elevate ROS levels and induce oxidative stress in cells. Moreover, nodosin could upregulate HMOX1, induce heme breakdown, release ROS, promote oxidative stress in cells, and induce apoptosis. 2) Excessive ROS production could lead to oxidative stress in cells. Oxidative stress was able to induce apoptosis. Finally, mitochondria and endoplasmic reticulum were destroyed in response to oxidative stress. Lysosomes removed damaged organelles and induced cells to undergo autophagy, inducing upregulation of autophagy markers LC3 and CTSL expression. Nodosin promoted cell death by inducing CRC cells to undergo apoptosis and autophagy. This figure is drawn by Figdraw (www.figdraw.com).

In conclusion, nodosin repressed CRC mainly through the induction of oxidative stress, triggering apoptosis, and autophagy. This study revealed the molecular mechanisms of nodosin against CRC: 1) Downregulation of *TRIB3* and upregulation of *OSGIN1* to induce oxidative stress in cells; 2) Elevation of *HMOX1* expression to induce apoptosis; 3) Upregulation of *CTSL* and *LC3* expression to trigger autophagy (As shown in Figure 7). However, we could not exclude other possible molecular mechanisms. This research provides solid experimental evidence for the suppression of CRC and greatly contributes to developing novel drugs for CRC therapy.

## Data Availability

Publicly available datasets were analyzed in this study. This data can be found here: The transcriptome data are uploaded on NCBI Sequence Read Archive and available *via* http://www.ncbi.nlm.nih.gov/bioproject/831009 (Bioproject ID: PRJNA831009).
